# Extrachromosomal DNA in Solid Tumors—Landscape, Immune Effects, and Resistance to Targeted Therapy

**DOI:** 10.32604/or.2026.075916

**Published:** 2026-04-22

**Authors:** Omar Badran, Siraj Attarya

**Affiliations:** 1 Department of Oncology, Emek Medical Center, Afula, Israel; 2 Technion Integrated Cancer Center, Faculty of Medicine, Technion, Haifa, Israel; 3 The Arab National Society for Health Research and Services, The Galilee Society, Shafa Amr, Israel

**Keywords:** Extrachromosomal DNA (ecDNA), double minutes, enhancer hijacking, immune-cold tumors, targeted-therapy resistance, liquid biopsy

## Abstract

Extrachromosomal DNA (ecDNA) constitutes a principal factor in the amplification of oncogenes and the progression of tumors in solid malignancies. This review synthesizes emerging mechanistic, genomic, and immunologic evidence across multiple tumor types, including glioblastoma, lung, breast, gastrointestinal, hepatobiliary, urothelial, prostate, gynecologic, pediatric, and head-and-neck cancers, with the goal of clarifying the role of ecDNA in immune escape and therapy resistance and outlining its translational implications for precision oncology. ecDNA comprises substantial acentromeric circular elements that serve as transcriptional hubs, modulate enhancer–promoter interactions, and undergo dynamic copy-number cycling, thereby fostering intratumoral heterogeneity and resistance to therapy. Recurrent oncogenic cargos, including epidermal growth factor receptor (EGFR), v-myc avian myelocytomatosis viral oncogene homolog (MYC), erb-b2 receptor tyrosine kinase 2, also known as human epidermal growth factor receptor 2 (ERBB2/HER2), and cyclin D1 (CCND1), are frequently located in ecDNA. They can interconvert with intrachromosomal homogeneously staining regions (HSRs) under treatment pressure. Emerging evidence links ecDNA to an immune-cold phenotype, characterized by downregulation of antigen presentation and decreased responsiveness to immune checkpoint inhibitors. We further emphasize diagnostic and translational methodologies that incorporate ecDNA detection through liquid biopsy and the spatial mapping of tumor topology. Finally, we propose a comprehensive clinical implementation framework that integrates ecDNA profiling, longitudinal monitoring, and immune microenvironment assessment to guide precision therapy. Gaining a deeper understanding of ecDNA biology has the potential to ultimately transform it from merely a prognostic biomarker into a targetable element within cancer therapy.

## 1 Introduction

Over the past century, cancer care has undergone a profound therapeutic evolution [1–4]. The earliest treatment paradigm relied almost exclusively on surgical resection, aimed at removing localized tumors before the onset of metastatic spread [5,6]. As systemic disease control emerged as a critical unmet need, the mid-20th century ushered in the cytotoxic chemotherapy era, providing the first tools capable of addressing disseminated malignancy, albeit with limited precision and substantial toxicity to healthy tissues [7–11]. The subsequent decades witnessed the advent of targeted therapies, enabled by the molecular characterization of oncogenic drivers, which transformed subsets of cancers, such as Epidermal Growth Factor Receptor (EGFR)-mutant lung cancer, Breakpoint Cluster Region–Abelson (BCR-ABL)–rearranged leukemia, and Human Epidermal Growth Factor Receptor 2 (HER2)-amplified breast carcinoma, from uniformly lethal diseases into conditions potentially controlled for years on treatment [12–17]. Yet targeted therapies fundamentally depend on the persistence of a dominant actionable alteration and are frequently undermined by acquired resistance driven by tumor plasticity, genetic diversification, and adaptive feedback signaling [18–21].

Immunotherapy has emerged within this continuum as a compelling “fourth pillar” of oncology, complementing surgery, chemotherapy, and targeted agents. It is grounded in the understanding that cancer progression reflects not only the biology of malignant cells but also the immune response escape [22–26]. Immune checkpoint inhibitors (ICIs), adoptive cell therapies, and cancer vaccines exploit the host immune system’s capacity to recognize and eliminate malignant cells, offering unprecedented and sometimes durable responses across diverse tumor types [27–30]. However, these benefits remain confined to a minority of patients, and biomarkers historically used to predict benefit, such as Programmed Death-Ligand 1PD-L1 expression, tumor mutational burden (TMB), and microsatellite instability (MSI), explain only a fraction of the observed clinical variability [31–35]. This gap has spurred intense interest in additional biological determinants that can refine patient selection and guide rational combinations [36–40].

Among these additional biological determinants, extrachromosomal DNA (ecDNA), large circular DNA elements that exist outside the chromosomes, has emerged as a key factor with profound implications for tumor evolution and immunotherapy outcomes [41–44]. The expanding genomic instability literature positions extrachromosomal DNA (ecDNA) as a central driver of oncogenic signaling, phenotypic heterogeneity, and immune evasion [45–48]. Unlike chromosomal amplifications, ecDNA provides tumor cells with a structurally distinct means of increasing oncogene dosage, rewiring enhancer–promoter interactions, and adapting dynamically to environmental and therapeutic pressures [48–51]. Mounting clinical evidence links ecDNA to aggressive disease biology, immune-excluded tumor microenvironments, and differential responses to targeted therapies and immunotherapy [52–54]. Against this backdrop, ecDNA has emerged as a candidate biomarker and a mechanistic framework to explain therapeutic non-response, especially in tumors that appear genomically “targetable” on sequencing yet behave as immunologically refractory or rapidly resistant [55–58].

A second and biologically distinct entity is extrachromosomal circular DNA (eccDNA). Unlike ecDNA, which spans hundreds of kilobases to megabases and clonally amplifies oncogenes, eccDNA comprises a heterogeneous collection of much smaller circular DNA fragments, typically less than 10 kb and often below 1 kb in length [59–63]. These molecules arise from routine genomic turnover, replication stress, and DNA repair processes, and are detected not only in cancer cells but also abundantly in normal tissues [64–68]. Accumulating data demonstrate that eccDNA can act as a potent trigger of innate immunity. When released into the cytosol, eccDNA activates DNA-sensing pathways, driving production of pro-inflammatory cytokines such as IL-6 and TNF-α [69–71]. These effects depend on the circular structure of eccDNA and can shape the tumor microenvironment, contributing to sterile inflammation, immune-cell recruitment, and, in some contexts, inflammation-linked carcinogenesis [72].

EcDNA, across various tumors, demonstrates heterogeneous distribution and locus-specificity, as exemplified by Human Epidermal Growth Factor Receptor 2 (ERBB2) and Epidermal Growth Factor Receptor (EGFR) in gastrointestinal cancers, the MYC family in small-cell lung carcinoma, pediatric embryonal tumors, and Platelet-Derived Growth Factor Receptor Alpha (PDGFRA)/EGFR in glioblastoma [46,53,73,74]. Recent studies reveal that ecDNA molecules do not act in isolation but instead cluster into nuclear “ecDNA hubs.” These hubs contain tens to hundreds of ecDNA circles and enable ecDNAs to cooperatively interact with regulatory enhancers, resulting in stronger and more frequent oncogene transcription. ecDNA hubs can include multiple distinct oncogenes and are found across cancer cell lines and primary tumors, providing a mechanism for elevated oncogene expression and accelerated tumor evolution [50,75–78].

Detecting ecDNA is most effectively approached as a layered workflow [79,80]. In tissue samples, short-read whole-genome sequencing (WGS), complemented by specialized amplicon callers such as AmpliconArchitect/AmpliconClassifier and JaBbA, can be employed to determine whether amplifications are circular (ecDNA-like) or intrachromosomal. Generally, high-confidence classification requires validation through orthogonal techniques, such as fluorescence *in situ* hybridization (FISH) or cytogenetics, to differentiate double minutes (DMs) from homogeneously staining regions (HSRs) [81–83]. Where feasible, the employment of long-read sequencing or optical mapping. Techniques such as High-throughput Chromosome Conformation Capture (Hi-C), Ribonucleic Acid (RNA) sequencing, and ATAC-seq facilitate the identification of enhancer hijacking and transcriptional hubs; however, they do not define the topology of such structures [78,79,81,84]. Conformational genomics approaches have provided direct experimental resolution of amplification topology. For example, Hi-C–based analyses of MYC- and PVT1-centered amplicons have demonstrated how circular extrachromosomal DNA architectures differ from linear intrachromosomal amplifications in their three-dimensional organization and enhancer–promoter connectivity, thereby illustrating the structural basis of enhancer rewiring in high-copy oncogene amplification [85,86]. Clinicians should be cognizant of the topology in reports, explicitly indicating whether ecDNA or HSR is present, including the confidence level, the predominant locus (and any co-amplified regulatory elements), the methods employed, and a recommended follow-up plan.

Scope of this review. We synthesize decision-grade, human-focused evidence and clarify where preclinical data inform practice. The review is structured into three parts: (i) a disease-level overview of ecDNA across tumors, emphasizing loci, functions, clinical signals, and liquid-biopsy applications; (ii) the relationship between ecDNA and anti-tumor immunity, including implications for immunotherapy selection and combinations; and (iii) mechanisms by which ecDNA contributes to resistance against targeted and biologic therapies, with pragmatic, mechanism-based strategies for treatment design and monitoring. Our objective is to provide a clear, clinician-ready framework for interpreting ecDNA results currently and for integrating ecDNA into future, biomarker-driven trials.

## 2 Methods

We conducted a clinician-oriented narrative review aimed at synthesizing decision-grade evidence regarding ecDNA across three domains: the disease-level landscape (including frequency, loci, functional programs, clinical outcomes, and liquid biopsy utility), the interface between ecDNA and anti-tumor immunity with implications for immunotherapy, and the mechanisms by which ecDNA contributes to resistance to targeted or biologic therapies. The purpose was to prioritize human data with definitive clinical endpoints, while utilizing mechanistic and preclinical reports to elucidate biology or to inspire testable interventions. Information sources included PubMed/Medical Literature Analysis and Retrieval System Online (MEDLINE), Scopus, and Web of Science from January 2000 through December 2025. We combined terms for ecDNA/Extrachromosomal Circular DNA (eccDNA), including “double minutes,” with cancer indications, assay modalities (e.g., AmpliconArchitect, JaBbA, long-read sequencing, CIRCLE-seq/Circle-Map, FISH, HSR), and clinical outcomes (objective response, PFS, OS, immunotherapy). We also screened reference lists of key primary studies and reviews. Grey literature was excluded except where a pivotal preprint was later peer-reviewed, in which case only the peer-reviewed version was used.

We accepted computational caller signatures of circular topology and favored studies with orthogonal validation (FISH, long-read, or breakpoint-level confirmation). Reports inferring circularity from copy-number alone were retained but labeled as putative. Decision-grade outcomes, such as objective response, progression-free survival (PFS)/overall survival (OS), and immunotherapy results, were prioritized, alongside immune-context readouts (e.g., Major Histocompatibility Complex (MHC-I), T-cell-inflamed), resistance mechanisms, and liquid-biopsy performance. Non-human models were included when they clarified mechanisms that aligned with human observations or supported a concrete therapeutic hypothesis. The study selection was conducted independently at both the title/abstract and full-text stages, with any disagreements resolved through discussion. Data extraction was performed using a standardized template, capturing tumor type and clinical context (primary, metastatic, post-relapse; adult vs. pediatric); ecDNA/eccDNA detection with locus-specific details, topology confidence, and evidence of regulatory co-amplification; functional programs such as enhancer hijacking, transcriptional hubs, Epithelial-to-Mesenchymal Transition (EMT), DNA damage response (DDR), metabolism, chromothripsis/reintegration, and amplification-linked extrachromosomal mutations; immune context; clinical outcomes including line of therapy and adjustment for confounding factors; liquid-biopsy matrix and assay performance; along with a concise, clinician-facing practice note. We indicated whether the evidence was patient-level or model-only and distinguished monotherapy from combination immunotherapy where feasible.

Considering the heterogeneity of study designs, we adopted a fit-for-purpose appraisal methodology rather than adhering to a conventional meta-analytic framework. Inclusion criteria comprised peer-reviewed human studies and clinically relevant preclinical investigations that evaluated ecDNA or eccDNA and provided extractable biological, genomic, or therapeutic outcome data. Exclusion criteria comprised non-oncologic ecDNA research, reviews lacking primary evidence, and duplicated datasets without additional analyses. All search strings, inclusion and exclusion criteria, and data extraction fields were recorded in a shared spreadsheet; overlapping datasets were consolidated into the most comprehensive analysis and cross-checked to prevent duplication. This work was not registered as a protocol, as it constitutes a narrative rather than a systematic review; nonetheless, we adhered to good-practice principles for transparent narrative syntheses and concentrated the discussion on human, outcome-linked evidence with explicit statements concerning certainty and clinical relevance.

## 3 Clinical Evidence of ecDNA across Tumor Types

Across many cancers, ecDNA frequently results in significant, conduct-targeted increases in the expression of cancer-related genes. We conducted an exhaustive review of human studies examining ecDNA within tumors. In this chapter, we systematically organize the findings by tumor type and employ a straightforward structure for each: the prevalence of ecDNA, the genes it encompasses, its impact on gene activity and tumor behavior, the implications for patients, including outcomes and treatment relevance, and the potential role of liquid biopsy (blood, urine, cerebrospinal fluid, bile) in testing and follow-up. Whenever beneficial, we highlight practical implications for clinicians and specify what to measure and how to measure it in routine reports. However, liquid biopsy–based monitoring of ecDNA remains an appealing yet technically difficult goal. While cell-free DNA (cfDNA) assays can reliably detect focal copy-number amplifications, they generally cannot determine the circular structure of ecDNA, and identifying circle-specific junctions in fragmented plasma DNA is still susceptible to false positives. Therefore, current liquid biopsy methods should be seen as providing indirect evidence of ecDNA levels rather than definitive topological information, highlighting the need for topology-aware assay development before clinical use.

**Figure 1:**
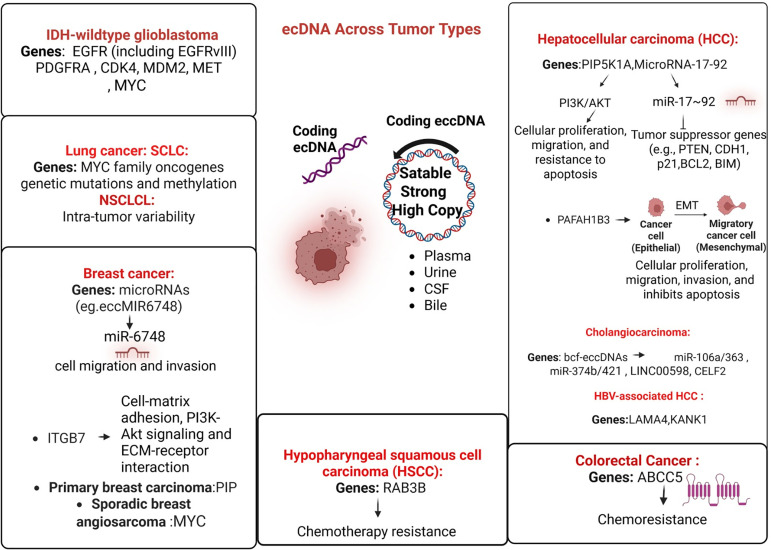
Extrachromosomal DNA (ecDNA) across tumor types and its clinical implications. IDH: Isocitrate Dehydrogenase, EGFR: Epidermal Growth Factor Receptor, PDGFRA: Platelet-Derived Growth Factor Receptor Alpha, CDK4: Cyclin-Dependent Kinase 4, MDM2: Mouse Double Minute 2 Homolog, MET: Mesenchymal-Epithelial Transition Factor, MYC: MYC Proto-Oncogene, Basic Helix-Loop-Helix Transcription Factor, SCLC: Small Cell Lung Cancer, NSCLCL: Non-Small Cell Lung Cancer, ITGB7: Integrin Subunit Beta 7, PIP: Prolactin-Induced Protein, CSF: Colony-Stimulating Factor, RAB3B: RAB3B, Member RAS Oncogene Family, PIP5K1A: Phosphatidylinositol-4-Phosphate 5-Kinase Type 1 Alpha, PI3K: Phosphoinositide 3-Kinase, AKT: Protein Kinase B, PTEN: Phosphatase and Tensin Homolog, CDH1: Cadherin-1 (E-Cadherin), BCL2: B-Cell Lymphoma 2, BIM: BCL2-Like 11 (BIM), PAFAH1B3: Platelet-Activating Factor Acetylhydrolase 1B Catalytic Subunit 3.

In glioblastoma, particularly in cases that are Isocitrate Dehydrogenase (IDH)-wildtype, research on brain tumors indicates that extrachromosomal DNA (ecDNA) is prevalent and frequently contains critical oncogenic drivers such as EGFR (including EGFRvIII), PDGFRA, Cyclin-Dependent Kinase 4 (CDK4), Mouse Double Minute 2 homolog (MDM2), MET, and Myelocytomatosis viral oncogene homolog (MYC) [87] (Fig. 1). Investigations tracking patient-derived tumors into neurospheres and orthotopic xenografts demonstrate that most of these circular amplifications are preserved [87]. However, MYC on ecDNA may newly emerge or expand upon recurrence [87]. This phenomenon reflects the unequal inheritance of ecDNA and the intense selective pressure for cells harboring multiple copies [87]. Under treatment with EGFR inhibitors, EGFRvIII on ecDNA may diminish, potentially giving an impression of target loss; however, it reappears following cessation of therapy, indicating rapid copy-number cycling rather than a true loss [87]. Spatial analyses of tumors reveal distinct regions with varied ecDNA lineages, for example, zones containing EGFR ecDNA (occasionally accompanied by short arm of chromosome 17 (17p)/Tumor Protein p53 (TP53) loss of heterozygosity) alongside regions with MDM2/MDM4 ecDNA, thereby illustrating the significant intratumoral heterogeneity and adaptability observed in glioblastoma [88]. In high-grade brain tumors (GBM), breakpoint-resolved sequencing and cytogenetic analyses provide direct evidence supporting the existence of circular amplicons (ecDNA) and transitions between ecDNA and HSRs [89]. The concept of amplification-linked extrachromosomal mutations elucidates how high-copy ecDNAs, such as those harboring EGFR or PDGFRA, may accumulate driver mutations and be subsequently lost or stabilized depending on selective pressures [53]. In clinical GBM samples, low-coverage whole-genome sequencing derived from routine FFPE tissue, combined with specialized computational algorithms, can reconstruct ecDNA structures, resolve breakpoints, and identify heterogeneous populations of wild-type and mutant EGFR ecDNAs [88,90]. FISH assays facilitate the validation of circular topology, thereby enabling the clear distinction between ecDNA and traditional intrachromosomal amplifications.

Lung cancer: In small-cell lung cancer (SCLC), extensive research converges on ecDNA as a primary mechanism underpinning oncogene amplification and tumor heterogeneity [91]. A comprehensive multi-omics analysis has shown that ecDNA often contains MYC family oncogenes and forms “transcription-amplifying units,” thereby linking ecDNA to tumor plasticity and reduced patient survival [91]. Notably, profiling circulating nucleosomes facilitates the non-invasive detection of ecDNA amplifications in plasma, thereby enabling genome-wide analysis of these alterations in SCLC and other malignancies [91]. A recent study utilizing a comprehensive collection of 51 patient-derived xenograft (PDX) models has demonstrated that, in SCLC, the acquired cross-resistance observed after relapse is consistently attributable to the amplification of MYC paralogs on ecDNA [92]. These preclinical models accurately reproduced the characteristic clinical features of SCLC, including the emergence of treatment-resistant disease after early relapse. Genomic and transcriptomic profiling has demonstrated that ecDNA harboring MYC family genes is frequently observed in relapsed, cross-resistant SCLC, a finding further validated in tumor biopsy samples from patients [92]. A focused review emphasizes that liquid biopsy profiling, which includes not only genetic mutations but also methylation, DNA fragmentation, and ecDNA readouts, may improve detection, subtyping, and treatment stratification in SCLC [92].

In non-small-cell lung carcinoma (NSCLC), prospective multi-region investigations demonstrate that ecDNA is prevalent, exhibits spatial heterogeneity, persists over time, and is associated with poorer prognoses [53,80]. An additional analysis disclosed that ecDNA was present in approximately 29% of NSCLC cases [93]. This was characterized by significant intra-tumor heterogeneity, often confined to regions, with a greater burden observed in advanced stages of the disease. This highlights its importance in the process of evolutionary diversification [93]. Research on liquid biopsy in lung adenocarcinoma (LUAD) substantiates the utilization of ecDNA/eccDNA as viable biomarkers. Analyses of plasma and serum specimens have identified cancer-specific eccDNA species, along with small panels that differentiate patients from controls with high Area Under the Curve (AUC) values, indicating their potential for early detection and monitoring [94]. Furthermore, tissues harvested from patients with early-stage lung cancer exhibited increased quantities and larger sizes of ecDNA molecules relative to matched normal tissues, with enrichment for driver genes. This finding further substantiates a biological and diagnostic signature from the initial stages.

In glioblastoma, circular amplicons often contain EGFR (including EGFRvIII), PDGFRA, CDK4, MDM2, MET, and MYC, promoting tumor heterogeneity and adaptive resistance. In lung cancer, extrachromosomal DNA harboring MYC family oncogenes plays a role in intratumoral heterogeneity in small-cell lung cancer (SCLC) and in the development of resistance in non–small-cell lung cancer (NSCLC). In breast cancer, ecDNA and eccDNA encode oncogenic microRNAs (e.g., eccMIR6748) and genes such as ITGB7, thereby promoting PI3K–Akt signaling, cell migration, and invasion; PIP-derived eccDNA and MYC amplification have also been documented. Hepatocellular carcinoma (HCC) exhibits recurrent extrachromosomal DNA (ecDNA) containing PIP5K1A and MIR17HG, which activate PI3K/AKT signaling pathways, induce epithelial–mesenchymal transition (EMT), and confer resistance to apoptosis. Meanwhile, cholangiocarcinoma is characterized by bile-derived ecDNA carrying miR-106a/363 and other regulatory elements that facilitate cellular proliferation and survival. Additionally, in colorectal cancer, amplification of ABCC5 on ecDNA confers resistance to chemotherapy. Similarly, hypopharyngeal squamous cell carcinoma (HSCC) acquires cisplatin resistance through eccDNA-mediated overexpression of RAB3B. The central illustration demonstrates the defining characteristics of eccDNA, its circular topology, capacity for autonomous replication, and notably high copy number, detected in plasma, urine, cerebrospinal fluid (CSF), and bile. This reinforces its potential utility as a biomarker for liquid biopsy.

Breast cancer research indicates that ecDNA is substantially more prevalent in tumor tissues relative to matched normal tissues. A subset of these molecules, which harbor microRNAs (eccMIRs), displays distinct expression patterns associated with signaling pathways pertinent to cancer development [95]. Notably, these over-represented eccMIRs are not merely passive entities; they actively contribute to the production of functional microRNAs within host cells [96]. For instance, eccMIR6748 increases miR-6748 levels, thereby facilitating breast cancer cell migration and invasion, underscoring a direct mechanistic role for eccDNA-encoded microRNAs in tumor progression [95]. Extensive analyses of breast cancer further indicate that approximately 26% of tumors exhibit ecDNA amplifications, with HER2 (ERBB2) being frequently implicated [53]. *In vitro* investigations demonstrate that aggressive triple-negative breast cancer (TNBC) cell lines, such as MDA-MB-231, exhibit a significantly higher abundance of ecDNA than Luminal-A cell lines, such as MCF-7, as assessed by fluorescence microscopy.

Furthermore, specific ERBB2 amplifications are observed as chromosomal HSRs, highlighting structural heterogeneity [96]. A diagnostic model created using these eccDNA features differentiates between malignant and normal breast tissue with an AUC of 0.83, indicating robust diagnostic potential [97]. Similarly, a prognostic model based on eccDNA achieves an AUC of 0.78, with repetitive-element annotations strongly correlated with prognosis. Interestingly, eccDNA characteristics do not differ significantly across various breast cancer subtypes or pathological stages. Another investigation indicates that in high-risk estrogen receptor-positive (ER+) and human epidermal growth factor receptor 2-positive (HER2+) breast cancers, a typical genomic architecture is observed, characterized by complex focal amplifications, including cyclic ecDNA [98].

Another study analyzes a meta-cohort of 1828 breast tumor samples (validated in an independent cohort of 2659) using whole-genome and transcriptome sequencing, demonstrating that breast cancers exist along a continuum delineated by three genomic “archetypes.” The high-risk estrogen receptor-positive (ER+) subgroup exhibits similarities to HER2-positive tumors and is characterized by intricate focal amplifications, including cyclic ecDNA. Induced by estrogen receptor (ER) signaling via R-loops and APOBEC3B activity, which are present even in pre-invasive lesions [99].

In contrast, triple-negative tumors exhibit genome-wide instability, characterized by tandem duplications and signatures resembling those of homologous repair deficiency. Conversely, typical-risk ER+ tumors are predominantly genomically stable. These archetypes are established early, remain conserved in metastatic disease, and influence the tumor microenvironment [100]. One study examined the structural modifications of the PIP gene on chromosome 7 in primary breast carcinomas and identified that a particular intragenic region of the PIP gene is not only amplified but also generates small extrachromosomal circular DNA (spcDNA) molecules in a subset of breast cancers. The amplified circular DNA includes exon 3, intron C, most of exon 4, and part of intron B of the PIP gene. This phenomenon, observed in 21.4% of breast tumors analyzed, suggests that spcDNA formation involving defined gene regions contributes to genetic instability in breast cancer and may serve as a target or marker for genomic variability in the disease [100]. Another study investigated the landscape and biological functions of eccDNA in breast cancer, emphasizing genes present on identified eccDNAs, including Integrin Subunit Beta 7 (ITGB7) (ITGB7). Using advanced sequencing and bioinformatics techniques, the authors identified 200 genes encoded by eccDNA that are enriched for pathways associated with actin cytoskeletal reorganization, focal adhesion, and the PI3K-Akt signaling cascade [100]. ITGB7 was notably emphasized for its roles in cell-matrix adhesion, membrane localization, and focal adhesion, as well as its connections with PI3K-Akt signaling and ECM-receptor interactions [100]. Notably, ITGB7 was significantly upregulated in breast cancer tissues and associated with the menopausal status of patients. The expression of this gene correlated with clinical outcomes, indicating its potential utility as a prognostic marker and highlighting its relevance for personalized treatment strategies. A concise report delineates an uncommon instance of sporadic breast angiosarcoma in a young woman, wherein elevated MYC amplification was identified on ecDNA via nanopore sequencing [101]. The sequencing analysis revealed ecDNA circles encompassing the MYC region, along with multiple structural variants within the 8q24 locus. The diagnosis was corroborated through methylation profiling and FISH.

**Figure 2:**
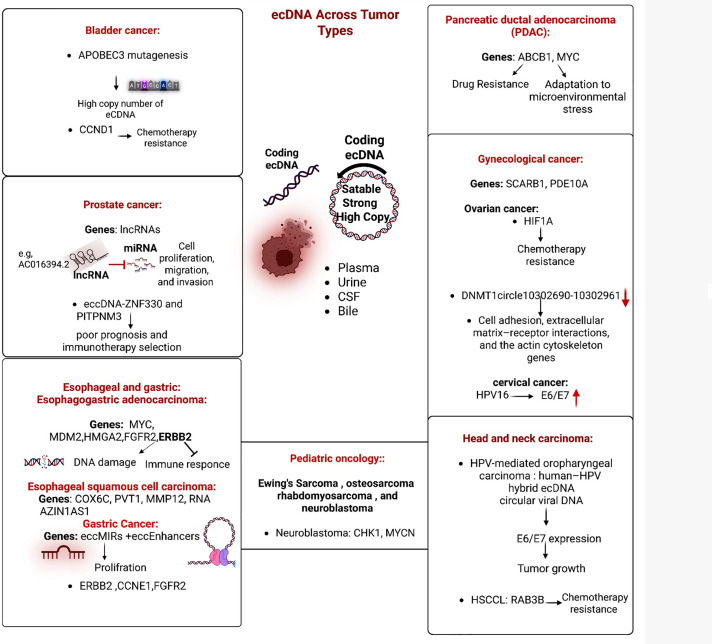
Pan-cancer representation of extrachromosomal DNA (ecDNA) and its biological and clinical effects. APOBEC3: Apolipoprotein B mRNA Editing Enzyme Catalytic Subunit 3, CCND1: Cyclin D1, ecDNA: Extrachromosomal DNA, lncRNAs: Long Non-Coding RNAs, ZNF330: Zinc Finger Protein 330, PITPNM3: Phosphatidylinositol Transfer Protein, Membrane-Associated 3, PDAC: Pancreatic Ductal Adenocarcinoma, MYC: MYC Proto-Oncogene, ABCB1: ATP-Binding Cassette Subfamily B Member 1, eccDNA: Extrachromosomal Circular DNA, SCARB1: Scavenger Receptor Class B Member 1, PDE10A: Phosphodiesterase 10A, HIF1A: Hypoxia-Inducible Factor 1 Alpha, HPV16: Human Papillomavirus Type 16, MDM2: Mouse Double Minute 2 Homolog, HMGA2: High Mobility Group AT-Hook 2, FGFR2: Fibroblast Growth Factor Receptor 2, ERBB2: Erb-B2 Receptor Tyrosine Kinase 2, RAB3B: RAB3B, Member RAS Oncogene Family, MYCN: MYCN Proto-Oncogene, CHK1: Checkpoint Kinase 1, CSF: Cerebrospinal Fluid.

Esophageal and gastric cancer: Longitudinal genomic profiling in Barrett’s esophagus demonstrates that ecDNA emerges early and undergoes positive selection during the progression to esophageal adenocarcinoma (EAC), characterized by increasing copy number and structural complexity [102] (Fig. 2). These ecDNA circles frequently include oncogenes and regulatory elements that facilitate gene amplification and transcription. In surveillance cohorts, ecDNA has been identified in pre-diagnostic Barrett’s biopsies, thereby supporting its use as a risk biomarker and justifying intensified endoscopic surveillance [102]. In a comprehensive series of esophageal adenocarcinoma (EAC) cases (n = 710), complex amplicons were identified and shown to originate through ecDNA and breakage–fusion–bridge (BFB) mechanism cycles; patient-derived organoids recapitulated ecDNA from primary tumors, and single-cell analyses captured ecDNA-driven clonal dynamics. Recurrently amplified oncogenes on ecDNA encompassed ERBB2, MYC, MDM2, and HMGA2 [103]. In a comparative whole-genome sequencing study involving 22 Esophageal squamous cell carcinoma (ESCC) and adjacent standard tissue samples, the researchers cataloged single-nucleotide variants/insertions and deletions; copy number variations/structural variations; and ecDNA, identifying five ecDNA events across four case patients [104]. These circles contained cancer-related loci, including Cytochrome c oxidase subunit 6C (COX6C), Plasmacytoma Variant Translocation 1 (PVT1), Matrix Metallopeptidase 12 (MMP12), and the oncogenic long non-coding Antizyme Inhibitor 1 Antisense RNA 1 (RNA AZIN1-AS1). Orthogonal analyses in TCGA-ESCA associated elevated expression levels of these ecDNA-localized genes with a reduced disease-free survival. Furthermore, a multivariable Cox model continued to identify ecDNA-borne amplifications as an independent prognostic factor, thereby supporting their potential utility for risk stratification in ESCC, despite the limited cohort size [104]. Across gastric cancers, ecDNA/eccDNA is prevalent and holds significant clinical relevance. Circle-seq studies demonstrate a substantial presence of eccDNA in gastric tumors, including circles containing enhancer elements (eccEnhancers) and microRNA precursors (eccMIRs) that increase miRNA production, suppress target genes, and facilitate cellular proliferation [105]. These findings support the concept that ecDNA/eccDNA function as a dynamic, adaptive reservoir. Structural-variant mapping in gastric cancer underscores recurring amplicon/SV hotspots at Erb-B2 Receptor Tyrosine Kinase 2 (ERBB2) (17q12) and Cyclin E1 (CCNE1) (19q12), frequently observed within chromothriptic contexts, aligning with ecDNA-mediated amplification in tumors characterized by chromosomal instability. Another report delineates an AGC case exhibiting Fibroblast Growth Factor Receptor 2 (FGFR2) amplification on ecDNA, along with a small Molecular Profiling-Based Assignment of Cancer Therapy (MONSTAR-SCREEN-2) cohort, in which four out of five FGFR2-amplified tumors exhibited dispersed FISH signals consistent with the described phenomenon of ecDNA. The authors suggest that FGFR2 on ecDNA is relatively common in FGFR2-amplified gastric cancer and may contribute to limited therapeutic efficacy and resistance to FGFR inhibitors [106].

Colorectal cancer (CRC): Comprehensive molecular profiling across the typical adenoma–carcinoma sequence in colorectal cancer (CRC) demonstrates that eccDNA and ecDNA elements notably expand during tumor progression. A significant ecDNA region on chromosome 8 (chr8: approximately 65–114 Mb) consistently exhibits an increase from adenoma to invasive carcinoma [107]. This observation has been extensively validated across independent patient cohorts and is associated with an unfavorable prognosis, thereby supporting its potential as both a biomarker and a therapeutic target.

Preclinical experiments suggest that targeting ecDNA formation, for example, with hydroxyurea, in combination with chemotherapeutic agents such as cisplatin, can effectively inhibit tumor growth [108]. Furthermore, modulation of MutS Homolog 3 (MSH3), a pivotal mismatch repair factor, diminishes double-stranded DNA breaks, impedes the formation of ecDNA and homogeneously staining regions, decreases Dihydrofolate Reductase (DHFR) gene amplification, and partially reinstates sensitivity to methotrexate [108]. These findings emphasize the importance of targeting repair pathways to mitigate ecDNA-mediated resistance and enhance clinical outcomes.

Pancreatic ductal adenocarcinoma (PDAC): ecDNA also plays a critical role in PDAC. Under paclitaxel treatment, PDAC models consistently develop multidrug resistance through the substantial induction of the drug-efflux transporter ATP-binding cassette subfamily B member 1 (ABCB1), partly via amplification of ABCB1 on extrachromosomal circles [109]. Short- and long-read sequencing techniques elucidate these structural features. Clonal experiments further demonstrate the potential for *de novo* ecDNA formation and rapid re-selection following therapeutic modifications. These findings underscore ecDNA dynamics as a potentially more feasible therapeutic target than direct ABCB1 inhibition.

Regarding lineage plasticity, ecDNA carrying MYC results in considerable dosage variation, thereby fostering intratumoral copy-number heterogeneity. This variation aids rapid adaptation to microenvironmental stress and, at the highest levels, correlates with squamous-like phenotypes and altered stromal dependencies [110]. The preservation of extrachromosomal MYC depends on continuous selective pressure, emphasizing how these circles transiently maintain aggressive states favored within particular niches [110].

Hepatocellular carcinoma (HCC) and biliary tract cancer: Recent genome-wide studies have established that exDNA (ecDNA/eccDNA) is highly prevalent in hepatocellular carcinoma (HCC) and plays an essential role in tumor biology and patient prognosis. Notably, a recurrent ecDNA of approximately three megabases derived from Chromosome 1, long arm (q), band 21 (1q21) has been identified in HCC [111]. This ecDNA contains the Phosphatidylinositol-4-phosphate 5-kinase type 1 alpha (PIK3C3) gene, whose amplification activates PI3K/AKT signaling, thereby promoting cellular proliferation, migration, and resistance to apoptosis [111]. Both functional genomic analyses and mechanistic assays show that Phosphoinositide 3-kinase (PIP5K1A) on ecDNA is linked to more aggressive tumor behaviors and a worse prognosis. This highlights its promising potential as a therapeutic target. Circle-seq profiling and related methodologies have further demonstrated that HCC tumors contain significantly more eccDNA than adjacent non-tumorous liver tissue [112]. Among these, eccDNAs encompassing the MicroRNA-17-92 Host Gene (MIR17HG locus), which encodes the oncogenic miR-17~92 microRNA cluster, are highly enriched in HCC and facilitate oncogenic processes by downregulating a range of tumor suppressors, including PTEN, CDH1, cyclin-dependent Kinase Inhibitor 1A (p21), BCL2, and BCL2-Like 11 (BIM) [112]. This mechanism facilitates tumor growth and invasion and is strongly correlated with unfavorable clinical outcomes, as evidenced by clinical datasets that associate eccDNA/gene copy number and gene expression with poor prognosis and earlier disease onset. Mouse model studies have demonstrated that small eccDNA fragments can carry mutations in tumor suppressor genes, exist independently of chromosomes, and contribute to intratumoral genetic heterogeneity by fluctuating allele frequencies over time [112]. This phenomenon persists even in the absence of ongoing genome editing, thereby facilitating tumor adaptation evolution. Furthermore, recent research has demonstrated that genes such as Platelet-Activating Factor Acetylhydrolase 1B catalytic subunit 3 (PAFAH1B3) may be present in both chromosomal and extrachromosomal DNA (eccDNA) states in hepatocellular carcinoma (HCC). The eccDNA form of PAFAH1B3 amplifies cellular proliferation, migration, and invasion and inhibits apoptosis, while also promoting epithelial–mesenchymal transition (EMT) [113]. These effects can be mitigated through targeted inhibition *in vivo*, thereby underscoring the physiological significance and potential therapeutic targeting of specific eccDNA molecules in HCC. Another study employed Circle-seq to investigate eccDNA in the bloodstream of patients diagnosed with HBV-associated HCC and liver cirrhosis. The researchers identified more than 100,000 unique eccDNAs in HCC and observed generally higher eccDNA abundance and diversity than in cirrhosis [114]. The majority of eccDNAs were less than 1000 base pairs in length. Bioinformatic analysis identified Laminin Subunit Alpha (LAMA4) and KN Motif and Ankyrin Repeat Domain-Containing Protein 1 (KANK1) as eccDNA-associated genes closely correlated with patient survival [114]. The findings indicate that blood-derived eccDNA profiles, particularly those involving LAMA4 and KANK1, may function as promising diagnostic and prognostic biomarkers for HCC.

The exploration of eccDNA within biliary tract malignancies, especially cholangiocarcinoma, is progressing swiftly. Recent findings have demonstrated the abundance of bile cell-free eccDNA (bcf-eccDNA) in malignant biliary strictures, with oncology samples showing not only diverse origins but also broader size distributions and several reproducible peaks validated in independent patient cohorts [115]. Functional investigations utilizing synthetic large eccDNAs have demonstrated that these circular DNA entities often encompass regulatory and oncogenic components, such as miR-106a/363 or miR-374b/421 [115]. These elements can influence oncogene expression, promote cellular proliferation, and inhibit apoptosis in both *in vitro* and *in vivo* settings. Clinically, bcf-eccDNA molecules retrieved from bile frequently harbor actionable cancer-specific mutations. RNA circles such as Long Intergenic Non-Protein Coding RNA 598 (LINC00598) and CUG-Binding Protein (CUGBP) Elav-Like Family member 2 (CELF2) have demonstrated high diagnostic accuracy (AUC > 0.9) for classifying malignant biliary strictures in both training and validation studies [115]. These findings position bile-eccDNA as a promising biomarker for liquid biopsy and as a mechanistically significant contributor to biliary carcinogenesis, potentially paving the way for enhanced diagnostic and therapeutic strategies in the management of cholangiocarcinoma and related diseases.

In bladder cancer, Apolipoprotein B mRNA Editing Enzyme Catalytic Subunit 3 (APOBEC3) mutagenesis and Cyclin D1 (CCND1) amplification on ecDNA contribute to genomic instability and chemotherapy resistance. In prostate cancer, ecDNA-associated long non-coding RNAs (lncRNAs), such as AC016394.2, regulate proliferation, migration, and invasion, while eccDNA carrying Zinc Finger Protein 330 (ZNF330) and Phosphatidylinositol Transfer Protein, Membrane-Associated 3 (PITPNM3) genes correlates with poor prognosis and immune-cold phenotypes. Pancreatic ductal adenocarcinoma (PDAC) demonstrates extrachromosomal DNA (ecDNA) containing ABCB1 and MYC, which facilitate multidrug resistance and adaptation to microenvironmental stress. In gynecological malignancies, ovarian cancer exhibits eccDNA carrying Scavenger Receptor Class B Member 1(SCARB1) and Phosphodiesterase 10A(PDE10A), as well as Hypoxia-Inducible Factor 1 Alpha (HIF1A)-associated chemoresistance; cervical cancers contain HPV16 episomes with rearranged E6/E7 oncogenes that promote carcinogenesis independently of chromosomal integration. In esophageal and gastric carcinomas, extrachromosomal DNA (ecDNA) commonly encompasses the MYC, MDM2, HMGA2, FGFR2, and ERBB2 genes, with eccEnhancers and eccMIRs contributing to cellular proliferation and immune suppression. Head and neck carcinomas, particularly HPV-mediated oropharyngeal and hypopharyngeal cancers, exhibit hybrid viral-human extrachromosomal DNA containing E6/E7 or RAB3B. The latter contributes to cisplatin resistance by activating autophagy. Finally, in pediatric tumors such as Ewing sarcoma, osteosarcoma, rhabdomyosarcoma, and neuroblastoma, ecDNA amplifications of MYCN and dependencies on CHK1 define aggressive disease and treatment resistance. The central schematic underscores the hallmark features of ecDNA, including its circular topology, autonomous replication, and high copy number. These characteristics are detectable across various biological fluids such as plasma, urine, cerebrospinal fluid (CSF), and bile, thereby highlighting its significance in cross-cancer diagnosis and therapy.

Bladder cancer, classified under urothelial carcinoma, acknowledges ecDNA as a pivotal factor in the tumor evolutionary mechanisms interacting with mutagenic processes. Longitudinal, multi-region genomic analyses indicate that Apolipoprotein B mRNA Editing Catalytic Polypeptide-like 3 (APOBEC3) mutagenesis occurs as an early, clonal event, while chemotherapy induces subsequent, subclonal mutation surges. In this context, genome-graph analyses reliably identify high–copy number circular amplicons [116]. Cyclin D1 (CCND1) is frequently amplified on ecDNA, and long-read assembly combined with functional modeling confirms that CCND1 circles persist, become increasingly complex following therapy, and actively contribute to chemotherapy resistance. This establishes a temporal interplay whereby early Apolipoprotein B mRNA Editing Catalytic Polypeptide-like (APOBEC) activity and subsequent stress induced by treatment collaboratively contribute to the biogenesis of ecDNA and confer a selective advantage [117]. Population-scale profiling further clarifies its prevalence and clinical importance. Among hundreds of tumor samples, ecDNA is identified in approximately one-third of cases, showing correlations with clonal diversification, multifocal disease, and an adverse prognosis.

Furthermore, ecDNA is associated with an immunosuppressive phenotype characterized by reduced expression of Major Histocompatibility Complex Class I (MHC-I) in malignant cells [55]. Notably, tumor-derived ecDNA can be detected with high specificity in urinary sediment DNA, thereby supporting a practical liquid-biopsy approach for surveillance and early diagnosis detection. Complementary cohort studies in bladder cancer demonstrate a substantial burden and notable structural heterogeneity of circular DNA, encompassing simple single-locus circles to intricate chimeric structures involving multiple chromosomes, which occasionally contain several oncogenes concurrently [116,117]. Large tumor-specific circles significantly influence overall transcriptomes; the ecDNA burden correlates with hypermutation, copy-number alterations, oncogene amplification, and patient outcomes [116,117]. Furthermore, matched urine samples accurately reflect tumor ecDNA signals.

Prostate cancer: Recent studies have demonstrated the clinical utility of genome-wide ecDNA and eccDNA profiling in prostate cancer [118]. A published ecDNA gene-based risk model supports stratifying patients by biochemical recurrence–free survival, achieving 1-, 3-, and 5-year AUCs of approximately 0.8. This performance surpasses that of traditional clinical risk assessment indices. This model focuses on six ecDNA-associated long non-coding RNAs (lncRNAs), including AC016394.2, the silencing of which in functional assays reduces cancer cell proliferation, migration, and invasion [118]. These findings suggest that ec-lncRNAs are essential contributors to the progression of prostate cancer and may serve as reliable prognostic biomarkers.

Furthermore, comprehensive investigations into urinary cell-free eccDNA in prostate cancer demonstrate that urine-derived circular DNA reflects the features of tumor-derived eccDNA [119]. These molecules primarily derive from unstable chromatin regions that are actively transcribed and characterized by double-strand breaks and R-loops. Genes associated with frequent eccDNA formation, including hepatocyte growth factor genes, are correlated with tumor progression and less favorable survival outcomes [119]. The experimental introduction of eccDNAs derived from prostate cancer–specific gene exons has demonstrated gene-regulatory effects, resulting in reduced proliferation and migration. This underscores the functional importance of small circular DNA molecules, regardless of whether their host genes are expressed. Another study comparing prostate cancer patients (both localized and metastatic) with individuals suffering from prostatitis and healthy controls found that plasma eccDNA levels are elevated in cancer cases, particularly in metastatic disease [120]. Using eccDNA feature profiles, machine learning models effectively distinguished cancerous from non-cancerous conditions, achieving an area under the ROC curve (AUC) of 0.91 in plasma samples with a neural network and 0.77 in urine samples with a random forest classifier [120]. eccDNA demonstrates potential as a non-invasive biomarker detectable in blood and urine for the diagnosis and monitoring of prostate cancer. A study investigated eccDNA in prostate adenocarcinoma by integrating Circular-seq data with The Cancer Genome Atlas (TCGA) datasets. The research identified 4290 dysregulated eccDNAs and 1981 amplified coding genes, subsequently pinpointing two eccDNA-amplified genes, ZNF330 and PITPNM3, to construct a two-gene risk model. The model stratified patients by prognosis (high-risk indicating poorer survival) and was correlated with distinct patterns of immune infiltration and immune escape [121]. Findings were validated on external datasets, and drug-sensitivity analysis suggested ten potentially beneficial therapies, supporting ZNF330 and PITPNM3 as candidate prognostic markers and providing a practical tool to guide PRAD prognosis and immunotherapy selection.

In renal cell carcinoma (RCC), ecDNA/eccDNA are gaining recognition as crucial factors contributing to genomic plasticity and heterogeneity, with RCC among the tumor types in which ecDNA amplifications have been observed [122]. Plasma eccDNA was analyzed using Tn5 transposon-sequencing NGS, which identified 8568 eccDNAs in patients compared to 8150 in healthy controls [123]. Although the overarching characteristics, such as length, gene annotation, and motif signatures, were comparable across the groups, the researchers discerned 701 eccDNAs with differential expression. Subsequently, they prioritized 25 candidates associated with tumor-related genomic regions [123]. These findings position plasma eccDNA as a promising non-invasive biomarker for detecting and monitoring ccRCC, while highlighting the need for validation cohorts and comprehensive diagnostic performance metrics before clinical implementation. Telomere maintenance via the Alternative Lengthening of Telomeres (ALT) pathway, often associated with extrachromosomal telomeric circles (t- and c-circles), is observed in a subset of renal cell carcinoma (RCC), particularly in telomerase-negative tumors [124]. This indicates the existence of an additional ecDNA-associated mechanism that could influence tumor evolution and patient prognosis. Overall, accumulating pan-cancer data indicate that ecDNA occurs in a significant proportion of human cancers and is associated with poorer outcomes. This context likely extends to RCC and underscores the importance of further translational research focusing on ecDNA-based diagnostics and therapeutic strategies. However, current data on ecDNA in renal cell carcinoma remain limited, thereby precluding robust mechanistic or clinically actionable conclusions currently.

Gynecological cancer: Plasma profiling in epithelial ovarian cancer demonstrates that circulating eccDNA levels are markedly elevated in patients compared with healthy individuals. Dynamic monitoring indicates that eccDNA levels generally increase during complete remission and decrease in partial response or relapse. The fold-change in eccDNA count (relative to baseline) yields an area under the curve (AUC) of approximately 0.71 for the purpose of distinguishing complete remission from partial response or relapse [125]. Furthermore, eccDNA mapping has identified Scavenger Receptor Class B Member 1 (SCARB1) and Phosphodiesterase 10A (PDE10A) as prognostic biomarkers, demonstrating AUCs of approximately 0.86 and 0.83, respectively [125]. Coverage analysis reveals enrichment of coding exons, and the chromosomal distribution corroborates risk stratification via top-gene annotation.

High-Grade Serous Ovarian Cancer (HGSOC): Multi-omics investigations in high-grade serous ovarian cancer (HGSOC) cell models have revealed a significant abundance of ecDNA, including HIF1A (Hypoxia Inducible Factor 1 Subunit Alpha), in cisplatin-resistant cell lines [126]. This indicates that pathways mediated by ecDNA, which are involved in the hypoxia response, DNA repair, and efflux mechanisms, are crucial in the development of resistance. Circle-Seq profiling uncovers approximately 160,000 eccDNA molecules, primarily less than one kilobase in length, enriched within non-coding repeat regions, with notable alterations observed on chromosome 21. Pathway analyses further associate eccDNA-related genes with mitotic spindle assembly, vascular permeability, and cellular differentiation. Comparative study of primary tumors and metastatic sites in advanced HGSOC reveals that DNA Methyltransferase 1(DNMT1) circle10302690-10302961 is consistently reduced in metastatic regions, with decreased abundance correlating with an adverse prognosis [127]. This suggests its potential utility as a biomarker for metastasis risk and clinical outcomes. A nine-gene signature, derived from eccDNA-associated genes and validated across TCGA and ICGC cohorts, effectively stratifies overall survival and differentiates immune infiltration patterns among risk groups (AUC approximately 0.67) [128]. Most corresponding eccDNAs range from 0 to 2 kilobases in length, frequently encompassing entire genes or gene fragments, and demonstrate enrichment in intronic, coding, and repetitive regions [128]. These eccDNAs influence cell adhesion, extracellular matrix–receptor interactions, and the actin cytoskeleton, which are essential components in ovarian cancer biology.

In cervical cancer, Human Papillomavirus type 16 (HPV16) can induce carcinogenesis by amplifying its genome as extrachromosomal episomes, without the necessity of chromosomal integration. Long-read whole-genome sequencing and cytogenetic techniques uncover extensive tandem arrays of both complete and truncated HPV16 genomes, exhibiting as “HPV superspreading” (distributed across multiple loci) or as multimeric episomes, occasionally resembling double minutes [129]. Approximately fifty percent of tumors harboring solely episomal HPV contain intact monomeric forms, whereas the remaining instances display rearranged and multimeric episomes. Of these, approximately 80% involve rearrangements in the E1/E2 region, leading to augmented E6/E7 expression analogous to that observed in tumors driven by integrated HPV [129]. These findings substantiate aberrant episomal replication and rearrangement as the predominant mechanism of HPV16-mediated carcinogenesis, operating independently of integration.

Pediatric oncology: An analysis of 1684 tumors found ecDNA in about 10% of cases, with much higher rates in more aggressive cancers such as Ewing’s Sarcoma (67%), osteosarcoma (47%), rhabdomyosarcoma (39%), and neuroblastoma (30%). The positivity for ecDNA correlates with decreased survival rates, and longitudinal studies demonstrate that ecDNA architectures evolve in conjunction with disease progression [130]. Extensive studies have shown that MYCN amplification serves as a significant predictor of unfavorable outcomes in cases of neuroblastoma. This amplification is commonly conducted on extrachromosomal circular DNA (ecDNA), rather than exclusively on linear chromosomes. Analyses of patient tumors and cell lines substantiate that MYCN copy number correlates strongly with ecDNA abundance, and that ecDNA amplicons exhibit distinctive structures and gene configurations [131]. A recent study used whole-genome and RNA sequencing techniques to show that CHK1, a kinase involved in the DNA damage response, is crucial for the survival of neuroblastoma (NB) cells with ecDNA-driven MYCN amplification [70].

Additionally, the study suggests that Checkpoint Kinase 1 (CHK1) inhibitors specifically target these cells. Moreover, ecDNA-related copy number heterogeneity encourages diverse neuroblastoma cell states, rapid transcriptional changes, and increased adaptability under treatment conditions. This emphasizes the clinical significance of ecDNA-specific treatment strategies. Foundational research further established that circular DNA structures in neuroblastoma harbor amplified MYCN in both primary and metastatic lesions, directly linking the presence of ecDNA to the disease’s aggressive nature [132]. Recent investigations into medulloblastoma demonstrate that ecDNA amplification occurs in 14%–18% of cases and is correlated with a significantly increased risk of relapse and reduced survival [133]. Single-cell imaging and multi-omic profiling demonstrate pervasive enhancer rewiring on ecDNA, significant heterogeneity in copy numbers, and the presence of multiple ecDNA lineages within tumors. Clustered Regularly Interspaced Short Palindromic Repeats (CRISPR) screening has identified functional distal enhancers on ecDNA as critical for cellular proliferation, thereby providing further insight into the mechanistic role of ecDNA as both a driver of tumor heterogeneity and a contributor to therapy resistance [134]. The role of ecDNA in pediatric tumors remains insufficiently characterized, underscoring the need for dedicated mechanistic and clinical studies.

Head and neck carcinoma: The study of head and neck cancers has increasingly identified a crucial role for ecDNA and eccDNA, which not only facilitate oncogenic transcription, especially in HPV-associated diseases, but also underpin cellular heterogeneity, therapeutic resistance, and novel mechanisms of immune evasion. Recent studies have demonstrated that, in cancers mediated by Human papillomavirus (HPV), tumors display notable increases in E6 star I (a splice variant of the HPV E6 oncogene) and other subtype-specific viral oncogene patterns, which are associated with the ecDNA subtype (hybrid vs. viral-only). Mechanistically, hybrid ecDNA positions the HPV oncogenes E6 and E7 (E6/E7) within an enhancer-rich environment, integrating somatic DNA with the HPV Late gene one enhancer (L1), which exhibits robust cis interactions [134]. Therapeutically, CRISPR interference targeting these enhancers and inhibition of Bromodomain and Extra-Terminal proteins (BET) domains selectively suppresses E6/E7 expression and tumor growth in ecDNA-positive models, providing an actionable epigenetic architecture [134]. These findings strongly support ecDNA-targeted treatments in HPV-driven oropharyngeal cancer. In Hypopharyngeal squamous cell carcinoma (HSCC), thousands of eccDNA molecules are present, ranging from 0.01 to 1000 kilobases. Specific genes, such as RAB3B, are transcribed exclusively from eccDNA rather than from linear DNA [135]. RAB3B was identified on a circular amplicon (chr1circle 46,219–52,682 kb) and validated by Polymerase chain reaction (PCR)/sequencing. Its expression is upregulated in cisplatin-resistant FaDu/DDP cell lines and patient-derived organoids. Functionally, Ras-related protein Rab-3B (RAB3B) on eccDNA promotes cisplatin resistance by inducing autophagy, as demonstrated by Monomeric red fluorescent protein–green fluorescent protein–LC3 autophagy flux reporter (mRFP-GFP-LC3) flux assays, Electron microscopy (EM), and gene manipulation experiments [135]. These results underscore the role of eccDNA in drug resistance in HSCC. However, evidence on the prevalence and functional significance of ecDNA in head and neck squamous cell carcinoma remains sparse, thereby limiting mechanistic understanding and clinical application.

From our perspective, across various diseases, ecDNA is not merely an obscure phenomenon but functions as a unifying factor influencing clinical outcomes. It consolidates therapeutically targetable drivers, redefines regulatory mechanisms beyond simple linear copy number variations, and enables swift, topology-dependent adaptation. The consistent pattern observed across glioblastoma, gastric, lung, bladder, and pediatric tumors, as well as HPV-OPC, indicates that the presence of ecDNA correlates with higher expression ceilings, increased spatial and temporal heterogeneity, and poorer outcomes, unless the amplified locus is precisely targetable and closely monitored. In practice, this emphasizes the importance of regular, topology-aware reporting (such as ecDNA vs. HSR, dominant loci, and co-amplified regulatory cargo) and supports serial measurements where feasible.

In parallel, advances in single-cell multiomics and spatial transcriptomics/proteomics now provide the resolution needed to dissect intratumoral ecDNA heterogeneity and lineage evolution, offering new avenues to monitor the dynamic regulatory programs and clonal architecture of cancer in real time. Diagnostics should transition from questioning “is there amplification?” to focusing on “what topology, what program, and how dynamic?” While short-read whole genome sequencing (WGS) paired with a validated variant caller can be beneficial in preliminary triage, orthogonal confirmation methods, e.g., FISH, optical mapping, or long-read sequencing, remain crucial for converting preliminary findings into decision-quality evidence, particularly when therapeutic choices rely on this information. Classical cytogenetic approaches remain informative in tumors with extensive structural complexity, complementing sequencing-based inference.

Liquid biopsies obtained from matrices such as plasma, urine, cerebrospinal fluid (CSF), and bile now facilitate longitudinal, locus-specific monitoring, even if they do not consistently elucidate structural topology. When employed alongside MHC-I and T-cell–inflamed scores, these methodologies enable “biological restaging” to predict resistance rather than merely document it retrospectively. Despite significant advances, the widespread clinical implementation of ecDNA-based diagnostics and routine topology-aware testing remains a work in progress; ongoing validation and the development of robust, cost-effective technologies are needed before these tools become routine parts of cancer management.

Therapeutically, the approach emphasizes treating biological factors rather than focusing solely on the genetic blueprint. For diseases positive for ecDNA, treatment strategies should prioritize combinations that regulate the dosage dynamics of Ribonucleotide Reductase (RNR) and Checkpoint Kinase 1 (CHK1), silence enhancer hubs through BET or super-enhancer strategies, or capitalize on emergent vulnerabilities such as ferroptosis in specific contexts of the Mitogen-Activated Protein Kinase (MAPK) pathway. Predefined on-treatment biomarker criteria should guide these strategies. Immunotherapy remains pertinent; however, monotherapy with checkpoint inhibitors should be de-emphasized in immune-cold, ecDNA-rich environments until antigen presentation is adequately restored or the tumor is effectively ‘warmed’. In summary: determine the topology, regulate the therapeutic strategy, reassess the biological state, and escalate treatment solely when data indicate that the tumor is prepared for such intervention.

## 4 ecDNA Shapes Tumor Immunity and Immunotherapy Response

**Figure 3:**
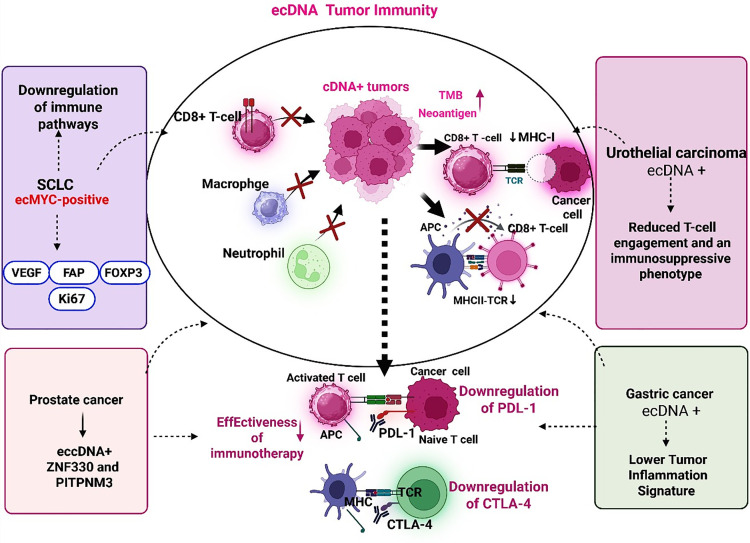
Immunological consequences of extrachromosomal DNA (ecDNA) across cancer types. ecDNA: Extrachromosomal DNA, TMB: Tumor Mutational Burden, MHC: Major Histocompatibility Complex, APCs: Antigen-Presenting Cells, TCR: T-Cell Receptor, CD8^+^: Cluster of Differentiation 8, SCLC: Small-Cell Lung Cancer, VEGF: Vascular Endothelial Growth Factor, FAP: Fibroblast Activation Protein, FOXP3: Forkhead Box P3, Ki67: Ki-67 Antigen, TIS: Tumor Inflammation Signature, ZNF330: Zinc Finger Protein 330, PITPNM3: Phosphatidylinositol Transfer Protein, Membrane-Associated 3, PD-L1: Programmed Death-Ligand 1, CTLA-4: Cytotoxic T-Lymphocyte–Associated Protein 4.

The relationship between ecDNA and immune escape is not merely descriptive; it unveils how genomic architecture can mechanistically reconfigure tumor–immune interactions, thereby determining which tumors remain perceivable or imperceptible to host immunity. Although individual studies describe ecDNA’s association with immune escape, collectively they elucidate a coherent biological framework: ecDNA predominantly restructures tumor–immune interactions by suppressing antigen presentation, modifying stromal composition, and facilitating the selection of immune-resistant clones. These converging mechanisms bear distinct therapeutic implications, some of which are already targetable, while others are still emerging. Although the number of studies on the relationship between ecDNA and immune modulation remains limited, there is nonetheless substantial evidence indicating that ecDNA plays a primary role in facilitating immune escape from cancer. One of these studies investigates the relationship between ecDNA and immune evasion in cancer, demonstrating that the presence of ecDNA in tumors is correlated with a substantial decrease in immune cell infiltration, particularly cytotoxic T cells, despite comparable tumor mutational burden (TMB) and neoantigen load relative to ecDNA-negative tumors (Fig. 3).

New evidence shows that tumors harboring ecDNA demonstrate hallmarks of immune evasion despite similar tumor mutational burden and neoantigen levels. These tumors exhibit reduced immune-cell infiltration, lower cytotoxic T-cell activity, and diminished expression of MHC class I and II antigen-presentation genes [136]. Their microenvironment is more immune-depleted, and less T-cell enriched, suggesting that ecDNA enables cancer cells to escape immune surveillance through impaired antigen presentation rather than reduced immunogenicity [136]. In summary, the formation of ecDNA may function as an intrinsic tumor mechanism facilitating immune evasion. The loss of antigen presentation is a direct pathway through which ecDNA-containing tumors become imperceptible to cytotoxic T cells, thereby establishing this pathway as central to immune escape. Among the immune phenotypes dependent on ecDNA, diminished antigen presentation is the most consistently validated and offers the greatest clinical potential, whereas stromal exclusion and metabolic vulnerabilities, although promising, remain predominantly preclinical. This understanding indicates that effective immunotherapeutic approaches for tumors rich in ecDNA may necessitate restoring antigen presentation alongside checkpoint inhibition. Of the immune pathways associated with ecDNA, re-establishing MHC expression appears to be the most immediately feasible, as it could re-sensitize tumors to checkpoint blockade.

In a comprehensive whole-genome sequencing (WGS) study of urothelial carcinoma at the population level, encompassing 595 samples, spatio-temporal heterogeneity of ecDNA was elucidated. The study correlated these findings with tumor progression, multifocality, and poor prognosis [137]. Notably, malignant cells exhibiting ecDNA positivity showed down-regulation of MHC-I at the single-cell level, consistent with decreased T-cell engagement and an immunosuppressive phenotype. This research supports a selection model in which ecDNA-bearing clones gradually proliferate, thereby influencing both heterogeneity and the immune microenvironment [137]. Furthermore, it demonstrates that detecting urinary sediment DNA can serve as a noninvasive approach to ecDNA analysis, thereby offering promising prospects for translational monitoring.

Nowhere is the impact of ecDNA on immune biology clearer than in small cell lung cancer (SCLC). EcDNA often facilitates the amplification of family paralogs (MYC, MYCN, and MYCL). This molecular event delineates a specific tumor subgroup distinguished by significant immunosuppression and resistance to therapy [138]. ecMYC-positive SCLC demonstrates widespread downregulation of immune-related pathways and a considerable decrease in T-cell infiltration within the tumor microenvironment. Spatial profiling and single-cell analyses reveal increased expression of proliferation markers (Ki67), angiogenic factors (VEGFA), fibroblast activation protein (FAP), and FOXP3-expressing regulatory T cell niches, underscoring both heightened tumor growth and immune exclusion [139,140]. Notably, treatment-naïve specimens already harbor dispersed MYC gains encoded by ecDNA, establishing early heterogeneity [140]. Functional studies demonstrate that inhibition of nucleotide metabolism effectively eradicates ecDNA and restores antigen presentation pathways, highlighting actionable therapeutic opportunities and vulnerabilities [140]. Together, these findings establish ecDNA as a crucial factor in immune evasion, tumor progression, and cross-resistance in SCLC, underscoring the clinical significance of targeting ecDNA-associated mechanisms to overcome immunotherapy resistance. In contrast, stromal remodeling and metabolic targeting continue to represent compelling yet predominantly preclinical opportunities that necessitate additional mechanistic validation.

A parallel line of evidence extends beyond ecDNA to implicate eccDNA in immune modulation [122]. In prostate cancer, the bipartite gene eccDNA risk model, which includes ZNF330 and PITPNM3, characterizes an immune-related phenotype in prostate adenocarcinoma [122]. The high-risk cohort demonstrates suboptimal immune infiltration and signs of immune dysfunction and exclusion, including an increased propensity for immune escape, as indicated by TIDE-like analyses, as well as a lower tumor mutational burden [122]. Importantly, this high-risk state is negatively linked to response to anti-PD-1/anti-CTLA-4 therapies, suggesting a more immunosuppressed tumor microenvironment less likely to benefit from checkpoint blockade [122]. In summary, eccDNA-related transcriptional amplification is associated with an immunologically “cold” profile, which is associated with a poorer prognosis and reduced sensitivity to immunotherapy.

Designed as a patient-selection biomarker for PD-1 therapy, ecDNA status identifies a subclass of gastric cancer characterized by features indicative of pembrolizumab insensitivity. An analysis presented at ASCO 2020 involving TCGA gastric cancer samples (N = 108) revealed that approximately 32% of the tumors tested positive for ecDNA, predominantly displaying mutual exclusivity with MSI-H (23%) [141]. ecDNA-positive tumors exhibited a lower Tumor Inflammation Signature and reduced PD-L1 expression [141]. Furthermore, Tumor Mutational Burden (TMB) was not elevated; only MSI-H tumors showed higher TMB levels, indicating an immune-cold phenotype unlikely to achieve sustained responses to PD-1 monotherapy. These findings support the development of clinical diagnostic methodologies for ecDNA detection and confirm ecDNA as a viable stratification in immunotherapy clinical trials. Although there are compelling associations, ecDNA-driven immune exclusion is unlikely to constitute a universal principle; viral tumors, hypermutated cancers, or neoplasms with significant pre-existing inflammation may supersede these effects. It is equally important to acknowledge that ecDNA-driven immune exclusion is context-dependent and may be diminished in hypermutated or virally driven tumors characterized by robust baseline inflammation.

From our perspective, ecDNA should be regarded less as a mere curiosity in tumor genomics and more as a clinically actionable axis in immune biology. Instead of questioning whether ecDNA correlates with immune exclusion, the pragmatic inquiry should focus on how to operationalize this knowledge: by prioritizing topology and immune profiling, favoring mechanism-aligned priming methods such as DNA Damage Response (DDR), metabolic, epigenetic, or driver/enhancer control strategies before escalating to ICIs, and monitoring biological dynamics, rather than solely anatomical features, over time through locus-aware liquid biopsies in conjunction with Major Histocompatibility Complex Class I (MHC-I) and T-cell–inflamed signatures. Caution should also be exercised to avoid overgeneralization, acknowledging the existence of exceptions, particularly in hypermutated or virally driven contexts, and recognizing that the causal relationship between ecDNA and immune suppression is likely bidirectional and context dependent. Nonetheless, the preponderance of evidence supports stratifying patients by ecDNA and designing clinical trials that target the induction of an inflamed state as an intermediate endpoint. Taken together, if we standardize ecDNA assays, harmonize immune readouts, and prioritize combination strategies to restore antigen presentation, ecDNA can evolve from merely a prognostic marker to a guiding instrument in therapy, helping avoid ineffective monotherapies and deliberately engineering tumors to become susceptible to immune checkpoint inhibition. Thus, the translational value of ecDNA lies not only in explaining resistance but in enabling mechanism-aligned immunotherapy strategies—restoring antigen presentation, reshaping stroma, and dynamically monitoring ecDNA content as tumors evolve.

This schematic illustrates how ecDNA alters the tumor–immune microenvironment by facilitating immune evasion and influencing immunotherapy efficacy. ecDNA-positive tumors demonstrate an increased tumor mutational burden (TMB) but show downregulation of Major Histocompatibility Complex (MHC) class I and II genes, thereby impairing antigen presentation and decreasing cytotoxic CD8^+^ T-cell infiltration. The microenvironment of such tumors is characterized by reduced numbers of macrophages and neutrophils, as well as reduced activity of antigen-presenting cells (APCs). Consequently, immune activation via T-cell receptor (TCR) engagement is attenuated. In small-cell lung cancer (SCLC), ecMYC-positive subtypes exhibit suppression of immune signaling pathways, accompanied by upregulation of proliferation and angiogenesis markers [such as Vascular Endothelial Growth Factor (VEGF), Fibroblast Activation Protein (FAP), Forkhead Box P3 (FOXP3), and Ki-67 Antigen (Ki67)], thereby demonstrating an immune-cold phenotype. Urothelial carcinoma harboring ecDNA exhibits decreased T-cell engagement and an immunosuppressive phenotype. Similarly, gastric cancers with ecDNA enrichment show a lower Tumor Inflammation Signature, suggesting a diminished likelihood of response to immune checkpoint inhibitors. In prostate cancer, eccDNA harboring ZNF330 and PITPNM3 is associated with reduced immunotherapy benefit and heightened immune exclusion. Collectively, ecDNA-mediated suppression of PD-L1 and CTLA-4 expression modifies immune checkpoints and limits the efficacy of immunotherapy, underscoring the need for combined strategies that restore antigen presentation and revitalize anti-tumor immunity.

## 5 ecDNA-Driven Resistance to Chemotherapy and Targeted Therapies

**Figure 4:**
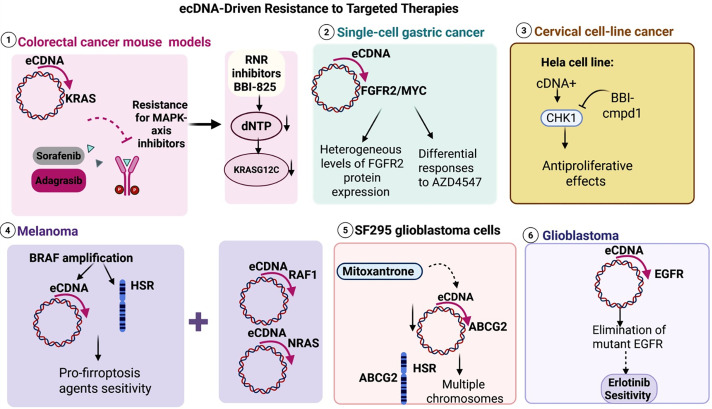
Mechanisms of extrachromosomal DNA (ecDNA)–driven resistance to targeted therapies across tumor models. KRAS: Kirsten Rat Sarcoma Viral Oncogene Homolog, ecDNA: Extrachromosomal DNA, MAPK: Mitogen-Activated Protein Kinase, FGFR2: Fibroblast Growth Factor Receptor 2, MYC: MYC Proto-Oncogene, AZD4547: AZD4547 (Selective Fibroblast Growth Factor Receptor Inhibitor), CHK1: Checkpoint Kinase 1, HSR: Homogeneous Staining Region, ABCG2: ATP-Binding Cassette Subfamily G Member 2, EGFR: Epidermal Growth Factor Receptor.

ecDNA has emerged as a critical factor in the evolution of cancer, resistance to therapeutic interventions, and immune evasion [53]. Unlike chromosomal amplifications, ecDNA offers tumors considerable genomic plasticity owing to its circular structure, accessible chromatin, and non-Mendelian inheritance patterns [142]. These features facilitate swift oncogene amplification, enhancer hijacking, and structural interactions between double minutes and homogeneously staining regions, thereby permitting cancer cells to adapt rapidly under selective pressures [142]. Recent experimental and clinical research increasingly indicates that ecDNA not only accelerates tumor progression but also impairs the efficacy of targeted therapies, chemotherapies, and immune checkpoint inhibitors. This section reviews significant findings from the literature, emphasizing mechanistic insights and translational applications that identify ecDNA as both a biomarker for resistance and a prospective therapeutic target (Fig. 4).

Patient and preclinical samples demonstrated focal amplification of the Kirsten Rat Sarcoma Viral Oncogene Homolog (KRAS) on ecDNA during adagrasib/sotorasib therapy. Signatures from metaphase/interphase Fluorescence *In Situ* Hybridization (FISH) and whole genome sequencing corroborated the circularization of the Kirsten Rat Sarcoma Viral Oncogene Homolog (KRAS) locus in resistant lesions [143]. In murine Kirsten Rat Sarcoma Viral Oncogene Homolog G12C (KRASG12C) models, recurrent tumors following KRAS inhibitor treatment exhibited KRAS extrachromosomal DNA, mirroring clinical observations. Resistance developed in the absence of consistent novel kinase-domain mutations, suggesting that increased dosage through ecDNA is adequate [143]. The data highlight a collective risk associated with ecDNA-enabled resistance to MAPK-axis inhibitors across the entire class. In practice, these findings support the development of combination therapies to prevent or limit the amplification of ecDNA. Moreover, a distinct study demonstrated that the selective RNR inhibitor (BBI-825) depleted deoxyribonucleotide triphosphate (dNTP) pools, thereby inhibiting ecDNA-enabled KRASG12C amplification during adagrasib treatment in colorectal cancer [144]. The combination therapy postponed the development of resistance and diminished proliferation in extended cultures. *In vivo*, co-administration eliminated KRAS ecDNA amplification and markedly hindered the growth of resistant tumors. Safety profiling demonstrated on-target selectivity across 78 assays [144]. The research identifies nucleotide supply as a crucial factor in ecDNA replication and advocates for RNR inhibition as both a prophylactic and therapeutic strategy against ecDNA-mediated resistance [144].

Subclones derived from single-cell gastric cancer exhibited divergent FGFR2/MYC ecDNA compositions, leading to heterogeneous levels of Fibroblast Growth Factor Receptor 2 (FGFR2) protein expression and differential responses to AZD4547 [145]. Resistant clones accumulated chimeric or enlarged ecDNA, or increased ecDNA quantities, thereby restoring FGFR2 signaling despite inhibitory interventions. Notably, the cessation of drug treatment reversed ecDNA states and re-sensitized the cells, underscoring the reversibility of these changes [145]. This study establishes a direct correlation between intraclonal ecDNA diversity and variability in receptor tyrosine kinase (RTK) output. It mechanistically links ecDNA dynamics with on-target resistance in the absence of novel point mutations. Glioblastoma cells acquired resistance to erlotinib by eliminating mutant EGFR situated on ecDNA, thereby reducing the abundance of the drug’s target. Subsequently, after drug holidays, mutant EGFR reemerged on ecDNA, restoring susceptibility, characterized by a reversible cycle of concealment and exposure. Single-cell analyses in both patients and experimental models confirmed this switching process on clinically relevant timescales [145].

The selection of the Human glioblastoma (GBM) cell line SF295 (SF295 glioblastoma) with Mitoxantrone resulted in a sequential amplification of the TP Binding Cassette Subfamily G Member 2 (ABCG2) on double minutes at lower dosages [146]. As the pressure increased, the double minutes diminished while HSRs emerged across multiple chromosomes, indicating reintegration. Northern and Western blot analyses, in conjunction with functional efflux assays, validated the overexpression of a functional transporter [146]. SKY karyotyping revealed extensive chromosomal rearrangements associated with the evolution of amplicons. These findings provide early, conclusive evidence of ecDNA-to-HSR transitions during the progression of chemoresistance. Additionally, they forecast recent observations of structural plasticity under drug-induced stress.

In murine models of colorectal carcinoma, amplification of KRAS on extrachromosomal DNA (ecDNA) promotes resistance to MAPK pathway inhibitors such as sorafenib and adagrasib. The concurrent use of the ribonucleotide reductase inhibitor BBI-825 reduces nucleotide pool availability, inhibits ecDNA replication, and thus delays the development of resistance. In cases of single-cell gastric cancer, ecDNA Harboring FGFR2 and MYC generates heterogeneous FGFR2 expression and varying responses to the FGFR inhibitor AZD4547, with swift reversibility of these ecDNA states upon cessation of treatment. In cervical cancer (HeLa) cells, ecDNA-positive clones demonstrate increased replication stress and a reliance on Checkpoint Kinase 1 (CHK1), making them highly susceptible to the CHK1 inhibitor BBI-cmpd1, which exhibits potent antiproliferative effects. In melanoma, BRAF amplification can occur as extrachromosomal (ecDNA) or chromosomal (HSR) forms, with ecDNA-containing cells exhibiting increased sensitivity to ferroptosis-inducing agents. In glioblastoma, exposure to mitoxantrone initially amplifies ABCG2 on extrachromosomal DNA (ecDNA), which is subsequently incorporated into multiple homogeneous staining regions (HSRs). Additionally, some glioblastoma models exhibit EGFR-bearing ecDNA that can be lost and regained during erlotinib therapy, exemplifying reversible copy-number cycling. Collectively, these observations illustrate that ecDNA functions as a dynamic genomic reservoir, facilitating rapid adaptation under therapeutic pressure, while concurrently unveiling vulnerabilities related to replication stress, nucleotide metabolism, and ferroptosis sensitivity.

Furthermore, secondary mechanisms, such as breakage-fusion-bridge (BFB)-mediated gains, also contribute to this phenomenon, underscoring the need to prevent ecDNA emergence or facilitate its elimination during de-escalation strategies [147]. The asymmetric inheritance of ecDNA leads to considerable heterogeneity in MYCN copy number at the single-cell level. This variability is associated with differences in RNA and protein expression levels, as well as phenotypic characteristics, with optimal fitness observed at moderate copy numbers [147]. During therapeutic interventions, tumors rapidly adjust ecDNA dosage, surpassing chromosomal amplification, with copy-number fluctuations reflecting treatment response and resistance in xenograft models and primary neuroblastomas [147]. Cells exhibiting senescence with low ecDNA levels serve as reservoirs for persistence, thereby supporting combination strategies aimed at targeting vulnerabilities across the spectrum of ecDNA dosages.

In HeLa cells on cervical cancer, CRISPR screening within an ecDNA amplification model has identified CHK1 as a specific dependency of ecDNA-bearing cells, consistent with observed elevated replication stress [148]. A potent and selective CHK1 inhibitor (BBI-cmpd1) exhibited enhanced biomarkers of replication stress and antiproliferative effects in ecDNA-positive cells compared to matched HSR or non-amplified controls. Additionally, this inhibitor effectively suppressed the growth of ecDNA-amplified tumors when administered orally *in vivo* [148]. These findings suggest that managing replication stress constitutes a vulnerability specific to ecDNA and support CHK1 inhibition as a promising therapeutic strategy for targeting ecDNA.

Ribonucleotide reductase (RNR), the rate-limiting enzyme in *de novo* deoxyribonucleotide synthesis, has been identified as a critical dependency in ecDNA-amplified cancers. In colorectal cancer models harboring KRAS^G12C mutations, KRAS amplification on ecDNA is a dominant mechanism of acquired resistance to KRAS^G12C inhibitors [148]. Preclinical studies demonstrate that the selective RNR inhibitor BBI-825 effectively suppresses ecDNA-enabled KRAS^G12C amplification, both preventing the emergence of resistance and inhibiting the growth of established KRAS^G12C ecDNA-amplified, drug-refractory tumors *in vivo* [148]. These findings support RNR inhibition as a synthetic-lethal strategy that targets the metabolic and replication demands imposed by ecDNA, rather than the oncogene itself, thereby delaying or reversing ecDNA-mediated adaptive resistance.

Emerging preclinical and early translational studies identify replication stress and excessive nucleotide demand as central liabilities of ecDNA-driven tumors, providing a mechanistically grounded framework to overcome ecDNA-mediated resistance to targeted therapies [149]. ecDNA frequently serves as the predominant platform for high-copy amplification of oncogenes, including resistance-conferring amplification of KRAS, EGFR, MYC, and MAPK pathway genes, thereby enabling rapid adaptive escape under therapeutic pressure [150].

Complementary evidence highlights checkpoint kinase 1 (CHK1) as a second, convergent vulnerability in ecDNA-driven cancers. Tumors harboring oncogene amplification on ecDNA exhibit heightened DNA replication stress and increased reliance on CHK1-mediated replication checkpoint signaling [151]. The oral, selective CHK1 inhibitor BBI-355 has demonstrated preferential antitumor activity in ecDNA-positive models, with enhanced induction of replication stress and durable tumor control across multiple preclinical systems [151]. Importantly, BBI-355 shows efficacy both as monotherapy and in rational combinations with targeted agents, including FGFR inhibitors in FGFR2 ecDNA-amplified gastric cancer and CDK4/6 inhibitors in CDK4 ecDNA-amplified sarcomas [151]. These combinations are particularly relevant in settings where targeted monotherapies promote further ecDNA-based amplification and resistance, thereby deepening tumor dependence on replication stress response pathways.

Together, these studies exemplify ecDNA as a primary regulator of adaptability: it amplifies driver and resistance genes, transitions between circular and chromosomal states, and adjusts copy number to optimize fitness during therapeutic interventions. This structural plasticity presents vulnerabilities, including replication stress (CHK1), nucleotide dependence (RNR), and susceptibility to ferroptosis, which may be leveraged therapeutically. For clinicians, it is essential to verify ecDNA topology, monitor locus-specific dynamics, and develop combination therapies that inhibit ecDNA formation and maintenance while targeting the amplified gene expression programs.

## 6 Future Directions

Moving forward, the field urgently requires the development of standardized methods for detecting and validating ecDNA. The current reliance on short-read whole-genome sequencing (WGS) without orthogonal validation techniques, such as fluorescence *in situ* hybridization (FISH) or optical mapping, often leads to misclassification of topology. The establishment of consensus pipelines that integrate computational callers with experimental verification, in conjunction with publicly available benchmark datasets, will help reduce heterogeneity and increase confidence in clinical applications. Prospective, topology-aware clinical trials constitute a crucial subsequent phase. Patients should be stratified by ecDNA vs. HSR status and the predominant oncogenic loci, with immune co-endpoints, such as MHC-I expression, T-cell–inflamed/IFN-γ signatures, and spatial Cluster of Differentiation 8 (CD8)/CAF density, defined at enrollment. Longitudinal sampling should be performed to monitor ecDNA dynamics over time and to enable more precise correlations between biological variables and therapeutic outcomes. Liquid biopsy methodologies hold promise for real-time patient monitoring. The advancement of topology-aware assays, such as those employing fragment omics, breakpoint phasing, or matrix-specific sampling techniques (including plasma, urine, cerebrospinal fluid, and bile), may enable serial tracking of ecDNA copy number and structural configuration. These tools could potentially be utilized for the detection of minimal residual disease and for treatment-guided “biological restaging”, thereby supplementing traditional RECIST criteria with an additional functional dimension. Beyond stratification, ecDNA has the potential to accelerate drug development. Incorporating ecDNA status into clinical trial eligibility criteria and adaptive trial designs could improve the detection of early signals, reduce the likelihood of failures at advanced stages, and inform rational escalation from monotherapy to combination strategies. Companion diagnostics aligned with ecDNA biology would support real-time go/no-go decision-making, enabling the prompt discontinuation of ineffective regimens and the early focus on promising mechanisms.

From a therapeutic perspective, it is essential to emphasize strategies that are aligned with fundamental mechanisms. The suppression of nucleotide supply, particularly RNR, along with the targeting of replication stress via CHK1 inhibitors, reveals promising vulnerabilities suitable for immediate drug development. Additionally, enhancer-targeted therapies, such as BET/SE inhibition, combined with ferroptosis induction, may function as context-specific adjuncts. Preclinical studies also indicate the potential to hinder ecDNA biogenesis and stability, for example, by targeting alternative end-joining mechanisms via DNA Polymerase Theta (POLQ) or by critical DNA repair pathways involving Breast Cancer Susceptibility Genes 1 and 2 (BRCA)– RAD51 Recombinase (RAD51). Moreover, therapies aimed at inducing ecDNA reintegration or promoting circle elimination could represent a novel class of therapeutic approaches. EcDNA dynamics may also inform dose timing and sequencing, such as initiating DDR priming before immune checkpoint inhibitors, positioning ecDNA not merely as a label but as a guiding principle for therapeutic strategy.

Ultimately, comprehending ecDNA plasticity, especially the transition between extrachromosomal and intrachromosomal (HSR) states, should be prioritized in translational research. Real-time observation of this process could guide dosing regimens and mitigate resistance arising from mode switching. The integration of spatial immunogenomic profiling techniques (such as imaging mass cytometry or single-cell multi-omics) will facilitate the correlation of ecDNA loci with immune phenotypes and stromal niches. Enrichment designs based on ecDNA, rather than all-comers immunotherapy trials, could reduce heterogeneity, increase statistical power, and facilitate meaningful interpretation of immune endpoints even in smaller cohorts. Pediatric tumors and rare cancers, which frequently exhibit elevated ecDNA burdens, constitute up-and-coming candidates for early interventional trials and the validation of liquid-biopsy–based surveillance.

By aligning ecDNA biology with immune monitoring and Mabey, we can use exosomes/liposomes containing anti-DNA fragments to improve targeting accuracy in therapeutic strategies, and the field can progress from descriptive investigations to actionable interventions. The establishment of open ecDNA/immune data repositories, the development of simulation models for ecDNA dosage dynamics, and the creation of companion diagnostics suitable for regulatory assessment will represent pivotal steps in transforming ecDNA from a biological curiosity into a valuable clinical instrument.

Despite rapid methodological advances, current approaches for detecting extrachromosomal DNA (ecDNA) remain subject to significant limitations that constrain biological interpretation and clinical translation. Most large-scale studies rely primarily on short-read whole-genome sequencing, which provides high sensitivity for focal amplifications but limited resolution of amplification topology. As a result, circular ecDNA structures can be challenging to distinguish from intrachromosomal amplifications such as homogeneously staining regions, particularly in the absence of systematic orthogonal validation. Computational ecDNA callers vary in their assumptions, thresholds, and performance characteristics, and their outputs are not always concordant across datasets. Experimental confirmation methods, including fluorescence *in situ* hybridization, metaphase spreads, or long-read sequencing, are therefore often required but are not uniformly applied, limiting cross-study comparability. In addition, most clinical sequencing pipelines are not optimized for ecDNA detection, and current liquid biopsy assays, while capable of identifying focal copy-number gains, cannot generally resolve amplification topology. Together, these constraints highlight the need for standardized, topology-aware detection frameworks and harmonized reporting practices before ecDNA can be reliably incorporated into routine clinical decision-making. Ultimately, the clinical adoption of ecDNA monitoring will necessitate the co-development of companion diagnostics in conjunction with therapeutic programs, ideally incorporated into regulatory submissions rather than conducted as retrospective analyses.

## 7 Limitations and Sources of Bias

The detection of ecDNA exhibits heterogeneity across various studies, characterized by differing call algorithms, threshold settings, and validation methodologies. Short-read whole-genome sequencing (WGS) may erroneously classify DNA topology, distinguishing ecDNA from homogeneously staining regions (HSR), in the absence of orthogonal confirmatory techniques such as fluorescence *in situ* hybridization (FISH), long-read sequencing, or optical mapping. Translational models, including patient-derived xenografts (PDX) and organoids, frequently overrepresent ecDNA-bearing clones relative to patient tumors, thereby inflating estimates of effect size. Many immunotherapy datasets are limited in size, retrospective in nature, and lack sufficient power to support histology-specific inferences. Longitudinal sampling efforts are inconsistent; thus, analyses of dynamic biological processes are complicated.

## 8 Conclusion

Extrachromosomal DNA (ecDNA) has emerged as a unifying mechanism of oncogene amplification, tumor evolution, and immune evasion across diverse contexts of cancer. Beyond serving as a marker of poor prognosis, it represents a tractable vulnerability through replication stress, metabolic dependencies, and enhancer rewiring. Integrating ecDNA status into clinical practice via prospective stratification, liquid biopsy monitoring, and biologic restaging offers the potential to refine patient selection and guide rational combination therapies. Ultimately, translating ecDNA biology into diagnostic and therapeutic strategies may reshape precision oncology and improve patient outcomes.

## Data Availability

Not applicable.

## Abbreviations

ecDNA: Extrachromosomal DNA
eccDNA: Extrachromosomal Circular DNA
ERBB2: Erb-B2 Receptor Tyrosine Kinase 2
EGFR: Epidermal Growth Factor Receptor
PDGFRA: Platelet-Derived Growth Factor Receptor Alpha
CAF: Cancer-Associated Fibroblasts
RNA: Ribonucleic Acid
Hi-C: High-throughput Chromosome Conformation Capture
MHC: Major Histocompatibility Complex
MHC-I: Major Histocompatibility Complex Class I
EMT: Epithelial-to-Mesenchymal Transition
IDH: Isocitrate Dehydrogenase
CDK4: Cyclin-Dependent Kinase 4
MDM2: Mouse Double Minute 2 homolog
MYC: Myelocytomatosis viral oncogene homolog
TP53: Tumor Protein p53
17p: Short arm of chromosome 17
AUC: Area Under the Curve
HMGA2: High Mobility Group AT-Hook 2
CCNE1: Cyclin E1
TCGA-CIN tumors: The Cancer Genome Atlas—Chromosomal Instability tumors
fSCNA+: Focal Somatic Copy Number Alteration positive
ABCC5: ATP Binding Cassette Subfamily C Member 5
DHFR: Dihydrofolate Reductase
MSH3: MutS Homolog 3
ABCB1 (MDR1): ATP Binding Cassette Subfamily B Member 1/Multidrug Resistance 1
PI3K/AKT: Phosphatidylinositol 3-Kinase/Protein Kinase B
PIP5K1A: Phosphatidylinositol-4-Phosphate 5-Kinase Type 1 Alpha
MIR17HG: MicroRNA 17-92 Cluster Host Gene
PTEN: Phosphatase and Tensin Homolog
CDH1: Cadherin 1
p21 (CDKN1A): Cyclin-Dependent Kinase Inhibitor 1A
BIM: BCL2 Interacting Mediator of Cell Death
BCL2: B-Cell Lymphoma 2
PAFAH1B3: Platelet Activating Factor Acetylhydrolase 1b Regulatory Subunit 3
LINC00598: Long Intergenic Non-Protein Coding RNA 598
CELF2: CUGBP Elav-Like Family Member 2
APOBEC3: Apolipoprotein B mRNA Editing Enzyme, Catalytic Polypeptide-like 3
CCND1: Cyclin D1
lncRNAs: Long Non-Coding RNAs
SYNCRIP: Synaptotagmin Binding Cytoplasmic RNA Interacting Protein
APOBEC: Apolipoprotein B mRNA Editing Catalytic Polypeptide-like
SCARB1: Scavenger Receptor Class B Member 1
PDE10A: Phosphodiesterase 10A
HIF1A: Hypoxia Inducible Factor 1 Subunit Alpha
HPV16: Human Papillomavirus type 16
E1/E2 (HPV): Early Protein 1/Early Protein 2
MB patients: Medulloblastoma patients
WNT: Wingless/Integrated
SHH: Sonic Hedgehog
HPV-OPC: Human Papillomavirus–Associated Oropharyngeal Carcinoma
BET inhibition: Bromodomain and Extra-Terminal domain inhibition
FaDu: Human hypopharyngeal squamous cell carcinoma cell line
FaDu/DDP: FaDu cisplatin-resistant cell line
mRFP-GFP-LC3: monomeric Red Fluorescent Protein—Green Fluorescent Protein—Microtubule-Associated Protein 1 Light Chain 3
RNR: Ribonucleotide Reductase
CHK1: Checkpoint Kinase 1
MAPK: Mitogen-Activated Protein Kinase
FAP: Fibroblast Activation Protein
FOXP3: Forkhead Box P3
DDR: DNA Damage Response
KRAS: Kirsten Rat Sarcoma Viral Oncogene Homolog
dNTP: Deoxyribonucleotide Triphosphate
BRAF: B-Raf Proto-Oncogene, Serine/Threonine Kinase
RAF: Rapidly Accelerated Fibrosarcoma
NRAS: Neuroblastoma RAS Viral Oncogene Homolog
FGFR2: Fibroblast Growth Factor Receptor 2
SF295: Human glioblastoma cell line SF295
ABCG2: ATP Binding Cassette Subfamily G Member 2 (BCRP—Breast Cancer Resistance Protein)
KB-V1: Human cervical carcinoma KB cell line variant with multidrug resistance
CD8: Cluster of Differentiation 8
POLQ: DNA Polymerase Theta
BRCA1/2: Breast Cancer Susceptibility Genes 1 and 2
RAD51: RAD51 Recombinase (PDAC) Pancreatic Ductal Adenocarcinoma
HCC: Hepatocellular Carcinoma
